# SigCS base: an integrated genetic information resource for human cerebral stroke

**DOI:** 10.1186/1752-0509-5-S2-S10

**Published:** 2011-12-14

**Authors:** Young-Kyu Park, Ok Sun Bang, Min-Ho Cha, Jaeheup Kim, John W Cole, Doheon Lee, Young Joo Kim

**Affiliations:** 1 Medical Genome Research Center, KRIBB, Daejeon 305-806, Korea; 2 Department of Bio and Brain Engineering, KAIST, Daejeon 305-701, Korea; 3 Department of Medical Research, KIOM, Daejeon 305-811, Korea; 4 Cogent Biotechnology Inc., Rockville, MD 20850, USA; 5 Maryland Stroke Center, Department of Neurology, Baltimore Veterans Affairs Medical Center and the University of Maryland School of Medicine, Baltimore MD 21201-1559, USA; 6 Genome Resource Center, KRIBB, Daejeon 305-806, Korea

## Abstract

**Background:**

To understand how stroke risk factors mechanistically contribute to stroke, the genetic components regulating each risk factor need to be integrated and evaluated with respect to biological function and through pathway-based algorithms. This resource will provide information to researchers studying the molecular and genetic causes of stroke in terms of genomic variants, genes, and pathways.

**Methods:**

Reported genetic variants, gene structure, phenotypes, and literature information regarding stroke were collected and extracted from publicly available databases describing variants, genome, proteome, functional annotation, and disease subtypes. Stroke related candidate pathways and etiologic genes that participate significantly in risk were analyzed in terms of canonical pathways in public biological pathway databases. These efforts resulted in a relational database of genetic signals of cerebral stroke, SigCS base, which implements an effective web retrieval system.

**Results:**

The current version of SigCS base documents 1943 non-redundant genes with 11472 genetic variants and 165 non-redundant pathways. The web retrieval system of SigCS base consists of two principal search flows, including: 1) a gene-based variant search using gene table browsing or a keyword search, and, 2) a pathway-based variant search using pathway table browsing. SigCS base is freely accessible at http://sysbio.kribb.re.kr/sigcs.

**Conclusions:**

SigCS base is an effective tool that can assist researchers in the identification of the genetic factors associated with stroke by utilizing existing literature information, selecting candidate genes and variants for experimental studies, and examining the pathways that contribute to the pathophysiological mechanisms of stroke.

## Introduction

Stroke is a heterogeneous complex disease that results from the interaction between genetic and environmental risk factors and has many well-established etiologies [1-5]. It is the second most common cause of death worldwide and is a major cause of acquired disability in survivors [5,6]. Environmental risk factors include smoking, alcohol intake, lack of physical activity, poor diet, and psychosocial stress/depression. Other established risk factors include hypertension, obesity, diabetes mellitus, and cardiovascular disease [2,5]. Several etiological risk factors including arteriovenous malformations, atherosclerosis, blood coagulation, sex hormone effects, hyperlipidemia, homocysteine, and inflammation have also been described [1,3-5].

Many genetic studies evaluating stroke pathophysiology and mechanisms have been conducted, resulting in hundreds of genes that appear to be associated with stroke. Most stroke-related genetic variants are causal markers derived using candidate gene approaches [7,8]. Stroke-related genetic loci have recently been identified implementing genome-wide association (GWA) studies [9,10]. Further, genetic variants related to other stroke etiologies, such as aneurysms [11,12] and diabetes [13], have also been reported.

To understand the contribution of the various risk factors to the mechanism of stroke, the genetic basis of each risk factor must be analyzed and integrated, in terms of biological function and pathway relationships. To this end, an efficient database system that integrates genetic variants and annotated information on stroke and its etiologies is required. Currently, there is no published or established database for this purpose, except the previous work by the author’s research group, StrokeBase [14]. In the previous work, cerebrovasular disease-related genes were collected from public databases, text-mining works with the goal to expand the functional information by using protein-protein interaction data and SNP-based genome-wide association study results. However, the system was substantially less informative due to an insufficient number of candidate genes, a lack of cross-links between the different types of the information, and inefficient user interfaces for the retrieval system.

Here we introduce SigCS base, an integrated genetic information resource for human cerebral stroke. SigCS base will allow researchers in molecular biology and genetics who study the causes and mechanisms of cerebral stroke to efficiently evaluate thousands of genomic variants and genes that are associated with stroke and its etiologies, as well as relevant annotated data from public molecular biology databases. By using the various retrieval features of SigCS base, researchers will be able to effectively refer to this information to select candidate genes and variants for their studies, to compare their genetic factors with previously reported results, and to examine the pathways that contribute to the pathological mechanism of stroke by comparing them between stroke and other etiologies.

## Methods

### Data source and processing

Online Mendelian Inheritance in Man (OMIM) [15] and Universal Protein Resource (UniProt) [16] were used to retrieve information on stroke- and etiology-related genetic variants; dbSNP [17] for SNP information; UCSC genome [18] for gene structure information, including transcript, exon/intron, coding region, and functional element data on SNPs; HUGO Gene Nomenclature Committee (HGNC) database [19] for standard gene names; Molecular Signatures Database (MSigDB) [20] for pathway and functional gene set information; and OMIM for literature information and PubMed links as raw data sources.

Eleven stroke etiologies/risk factors were selected: hypertension [1-3,21,22], obesity [2,3,23]—known to contribute to both ischemic and hemorrhagic stroke [5]—type 2 diabetes mellitus [2,3,5,22], hyperlipidemia [3,5,22], atherosclerosis [3,24], blood coagulation [3,4,25], vascular inflammation [4,26,27], estrogen effect [28,29], hyperhomocysteinemia [4,25,30] for ischemic stroke, intracranial aneurysm [1,31], and arteriovenous malformations [1,31,32] for hemorrhagic stroke. Then, to construct a variant and gene set, we downloaded stroke- and stroke etiology-related records in XML format from the OMIM database through a refined keyword search (Table 1) and parsed them.

**Table 1:** Keywords used to retrieve OMIM database for stroke and its etiology related records

Stroke or its etiology	Keywords for OMIM search	Etiology Code
Atherosclerosis	Atherosclerosis; Atheroscleroses; Fatty Streak, Arterial; Arterial Fatty Streak; Streak, Arterial Fatty; Atheroma; Atherogenesis	ATSC
Blood coagulation	Blood Coagulation; Blood Clotting	COAG
Cardiovascular Disease	Cardiovascular Disease	CAVD
Diabetes mellitus, type 2	Ketosis-Resistant Diabetes Mellitus; Non-Insulin-Dependent Diabetes Mellitus; Non Insulin Dependent Diabetes Mellitus; Noninsulin Dependent Diabetes Mellitus; Type 2 Diabetes Mellitus; Slow-Onset Diabetes Mellitus; Slow Onset Diabetes Mellitus; Stable Diabetes Mellitus; Diabetes Mellitus, Type II; Maturity-Onset Diabetes Mellitus; Maturity Onset Diabetes Mellitus; MODY; NIDDM; Adult-Onset Diabetes Mellitus	T2DM
Estrogen effect	Estrogen	ESTR
Homocysteine	Homocysteine	HCYS
Hyperlipidemia	Hyperlipidemia; Hyperlipemia; Hyperlipidemia; Lipidemia; Lipemia	HLIP
Hypertension	Hypertension; High Blood Pressure	HTNS
Vascular inflammation/Vasculitis	Inflammation	INFL
Intracranial arteriovenous malformations (AVM); Aneurysm	Intracranial Aneurysm; Basilar Artery Aneurysm; Cerebral Artery Aneurysm; Berry Aneurysm; Brain Aneurysm; Cerebral Aneurysm; Intracranial Mycotic Aneurysm; Communicating Artery Aneurysm; Intracranial Arteriovenous Malformation; AVM Intracranial; Intracranial AVM; Cerebral Arteriovenous Malformation; Ruptured Intracranial Arteriovenous Malformation	IAVM
Obesity	Obesity	OBES
Stroke	Stroke; Cerebral Stroke; Brain Vascular Accident; Cerebrovascular Apoplexy; Cerebrovascular Stroke; CVA; Cerebrovascular Accident; Apoplexy	STRK

To enhance the variant information, we extracted variant, phenotype, and literature data from the downloaded flat files of UniProt. To add the functional significance of each variant, we extracted gene structural annotations and SNP functional annotations from a downloaded flat file from UCSC genome. We extracted the flank sequences of SNPs from the dbSNP flat file to help researchers' experimental studies.

All gene symbol data was converted to the standard gene symbols of HGNC to ensure compatibility between the datasets.

### Pathway analysis

To attain insight into the biological functions and pathological mechanisms of stroke and its etiologies, we analyzed the biological pathways that significantly overlapped with the curated stroke and etiology gene sets. For this, we counted common genes between the stroke and etiology gene sets and the genes in known canonical pathways in MSigDB [20] and performed statistical testing to assess the significance of the overlaps. An one-tailed version of Fisher’s exact test (http://en.wikipedia.org/wiki/Hypergeometric_distribution) based on the hypergeometric distribution (k, K, n, N) of k overlapping genes in a user gene set of n genes and the pathway having K genes in a total gene space of N genes was used for the statistical test. An adjustment for multiple testing followed by false discovery rate evaluation was performed. For these analyses 639 pathways with including 5385 genes from BioCarta (http://www.biocarta.com/), KEGG (http://www.genome.jp/kegg/), and Reactome (http://www.reactome.org/) databases were implemented.

### Database implementation

A relational database using MySQL was constructed implementing a web retrieval system in the PHP language. To manage and service all the derived information an Apache web server on a Linux platform (Dell PowerEdge Server with 2 2.66 GHz Intel Quad Core Xeon CPUs, 32 GB memory, and 6TB SAS hard drive) was implemented.

## Results

The current version of SigCS base documents 1943 non-redundant genes with 11472 genetic variants and 165 non-redundant pathways (Table 2). The web retrieval system of SigCS base consists of two principal search flows: 1) a gene-based variant search using gene table browsing or keyword search, 2) a pathway-based variant search using pathway table browsing. SigCS base is freely accessible at http://sysbio.kribb.re.kr/sigcs.

**Table 2:** Statistics on the information in SigCS base

No.	Stroke or Etiology	# of Genes	# of Variants	# of Sig. Pathways^*^
1	Stroke	236	1609	18
2	Atherosclerosis	200	891	19
3	Cardiovascular disease	143	501	9
4	Coagulation, blood	47	732	19
5	Estrogen	353	851	6
6	Hypercysteinemia	90	118	2
7	Hyperlipidemia	102	333	10
8	Hypertension	521	2177	13
9	Intracranial arteriovenous malformations (including intracranial aneurysm)	60	182	11
10	Inflammation	696	1354	94
11	Obesity	412	1121	27
12	Diabetes mellitus, type 2	208	1603	19
Total	Stroke + All etiologies	3068(1943)^†^	11472	247(165)^†^

* Pathways with the FDR q-values of less than 0.05.

† Numbers in ( ) are counts for non-redundant genes or pathways over the etiologies.

### User interface

**Figure 1:**
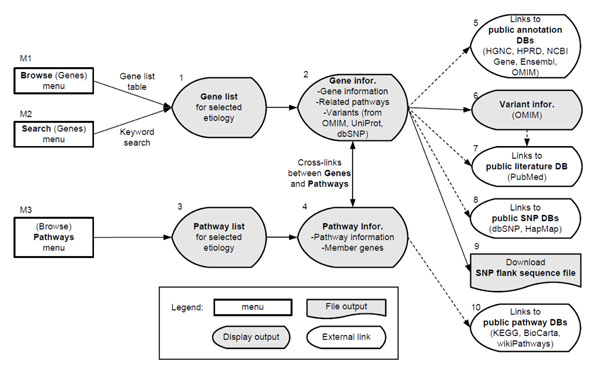
**Two main search flows in SigCS base.** Users can retrieve stroke and its etiology related genetic variant and biological pathway information through the two principal search flows of SigCS base: a gene-based variant search (M1 or M2 →1→2→5, 6, 7, 8, 9 or 4) and a pathway-based variant search (M3→3→4→10 or 2).

Users can retrieve genetic variant and biological pathway information that are related with stroke and its etiologies in SigCS base through its two principal search flows including: a gene-based variant search (M1 or M2 →1→2→5, 6, 7, 8, 9 or 4 in Figure 1) and a pathway-based variant search (M3→3→4→10 or 2 in Figure 1). The former uses genes as an entry point of variant information through a gene table browse function or keyword search. The latter uses pathways as an entry point of variant information through a pathway table browse function. The two search flows connect in the middle of the search flows using cross-links between genes and pathways.

#### Gene-based variant search

**Figure 2:**
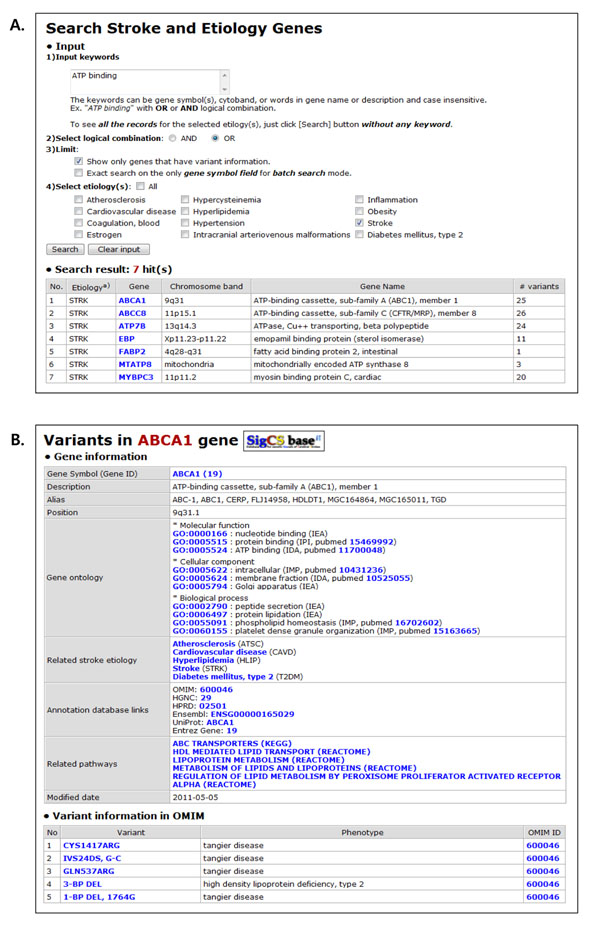
**Screenshots for the keyword search page and a gene and variant information page in SigCS base web interface.** The keyword search for the stroke and its etiology related genetic variant information in SigCS base is available at the keyword search page. Some useful user options including simple logical combination, limitation of search in terms of the existence of variants, batch search, and a multiple selection of etiologies to be searched are provided (Panel A). Gene and variant information page shows a gene information table, an OMIM-originated variant table, a UniProt-originated variant table, and a dbSNP-originated variant table (Panel B). In the figure, only the gene information table and an OMIM-originated variant table are shown.

In this search flow methodology, users can retrieve genetic variant information that is related to stroke and its etiologies through a table browse or keyword search function. To do so, at first users can peruse the resulting gene list in table format by choosing the ‘Browse’ menu (M1 in Figure 1) or search the genes by keyword search on the gene symbols or gene descriptions stored in SigCS base at the keyword search page (A in Figure 2) by choosing the ‘Search’ menu (M2 in Figure 1).

After examining the gene list and description by the gene table browsing or keyword search, users can click a gene symbol of interest and retrieve the gene information page, comprising a gene information table, an OMIM-originated variant table, a UniProt-originated variant table, and a dbSNP-originated variant table (B in Figure 2).

**Figure 3:**
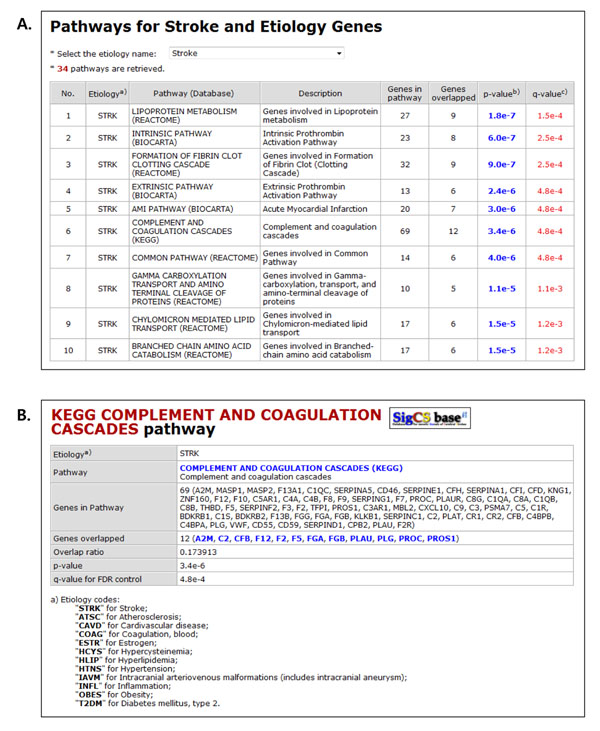
**Screenshots for the pathway browse page and a detailed pathway information page.** Users can retrieve pathways and gene sets contributable for stroke or each etiology at the pathway browse page (Panel A) and get the detailed pathway information (Panel B) by clicking the link on the p-value of a pathway.

The gene information table lists basic data on the gene, hyperlinks to functional annotation databases, and data on the biological pathway to which the gene is assigned. The hyperlinks connect to public annotation databases, including Entrez Gene [33], HGNC, OMIM, Ensembl [34], and HPRD [35]. Clicking the link on a pathway name generates a pathway information page that contains detailed gene pathways, hyperlinks to the source pathway annotation database, a list of other genes that are assigned to the same pathway, and p-values for pathway assignments (B in Figure 3).

The OMIM-originated variant table displays the variants in the selected gene and related phenotype information. Links on the variant name generates a page that contains detailed variant information and shows experimental data, phenotype data, and references on that variant.

The UniProt-originated variant table shows nonsynonymous variants, ordered by amino acid coordinates in the protein, for the selected gene, as well as amino acid pairs that correspond to the variants, related phenotypes, and PubMed links.

The dbSNP-originated variant table lists the SNPs, based on the genomic structure of the gene, ordered by coding strand. The structure of the gene includes the 2K upstream base pairs, exons, introns, 5’ UTR, 3’ UTR, and 500 base-pair downstream region. Each SNP information block is composed of a genomic coordinate (in base pairs) on the chromosome, the rsID, mutation function, and links to the dbSNP and HapMap databases [36]. Researchers can evaluate the function or usability of each SNP in experimental studies using the provided information on function, detailed SNP information from dbSNP, and linkage disequilibrium and tag SNP data from HapMap. A SNP flank sequence file can be downloaded selectively for further study.

#### Pathway-based variant search

In this search flow, users can retrieve pathways and gene sets that statistically analyzed to contribute functionally to stroke or each etiology through the ‘Pathways’ menu (M3 in Figure 1). This flow is helpful in understanding the role of genes or variants when the relationships between the genes and stroke or its etiologies are obscure. By selecting the combo box at the top of the table at the pathway browse page, a table lists biological pathways that are statistically related to stroke or its etiologies, ordered by p-value and q-value (A in Figure 3). The link on the p-value generates a page that contains detailed pathway information, hyperlinks to the source pathway annotation database, and a list of other genes that are assigned to that pathway (B in Figure 3). The links on the gene symbols in the overlapping gene list provide crosslinks to gene information pages; thus, more efficient gene variant searches can be undertaken.

## Conclusions

SigCS base is an effective user-friendly tool designed to assist researchers in the identification of the molecular and genetic factors associated with stroke. As described, SigCS base comprehensively implements numerous genetic databases, including OMIM [15], UniProt [16], dbSNP [17], UCSC genome [18], HGNC database [19], MSigDB [20], providing detailed variant, gene and pathway information to maximize the accuracy of the results produced. These results are further enhanced by including data regarding established stroke etiologies and risk factors, including: hypertension, obesity, type 2 diabetes mellitus, hyperlipidemia, atherosclerosis, blood coagulation, vascular inflammation, estrogen effects, hyperhomocysteinemia, intracranial aneurysms, and arteriovenous malformations.

While highly useful in its current form, SigCS base will be updated periodically as the molecular and pathophysiological understanding of stroke grows with regard to function and as more database content becomes available. For example, in the next SigCS base update, PubMed will be used as the raw data source for variant information rather than OMIM as currently implemented. While OMIM is accurate, the information content is often delayed as compared to PubMed. We also plan to employ text-mining techniques and manual curation to extract gene and variant information, in an effort to retrieve greater and more precise content. Our planned SigCS base content updates will not only provide an improved list of stroke etiologies and risk factor data, but will also increase accuracy by merging certain etiologies that share pathological causalities and adding new etiologies that contribute to stroke. The genetic variants, genes and pathway relationships as derived from more reliable etiologies will provide a more accurate understanding and insight towards the mechanisms of stroke. Users' comments and suggestions for additional features of interest are welcomed.

## Competing interests

The authors declare that they have no competing interests.

## Authors' contributions

YP, DL, and YJK conceived the study and designed functions. YP and YJK implemented core programs and web interfaces. OSB, MC, JK, and JWC selected etiology, curated data and constructed database. DL and YJK directed the investigation. YP, DL, and YJK wrote the manuscript. All authors read and approved the final manuscript.
